# [μ-4-Benzoyl-1-(1-oxido-2-naphthyl­carbon­yl)thio­semicarbazidato(4−)]bis­[pyridine­copper(II)]

**DOI:** 10.1107/S1600536809040951

**Published:** 2009-10-17

**Authors:** Wen Zhang, Jin-Ping Gao, Xue-Feng Shi, Da-Cheng Li

**Affiliations:** a College of Chemistry and Chemical Engineering, Liaocheng University, Shandong 252059, People’s Republic of China

## Abstract

In the title dinuclear complex, [Cu_2_(C_19_H_11_N_3_O_3_S)(C_5_H_5_N)_2_], the two Cu^II^ centers have different coordination environments, *viz.* N_2_OS and N_2_O_2_, each exhibiting a distorted square-planar geometry. π–π inter­actions between the aromatic rings of neighbouring complexes [centroid–centroid distance = 3.856 (5) Å] link pairs of mol­ecules into centrosymmetric dimers, which are further packed into stacks along the *b* axis with relatively short Cu⋯Cu separations of 3.482 (1) Å. Weak inter­molecular C—H⋯N hydrogen bonds help to consolidate the crystal packing.

## Related literature

For details of the synthesis, see: Chen *et al.* (2007 ▶). For pharmacological properties of complexes of acyl­thio­semicarbazides, see: Wei *et al.* (1995 ▶).
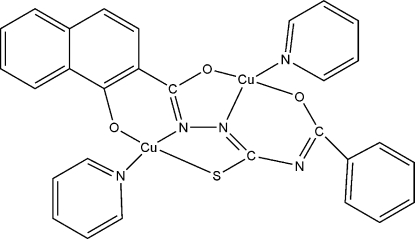


## Experimental

### 

#### Crystal data

[Cu_2_(C_19_H_11_N_3_O_3_S)(C_5_H_5_N)_2_]
*M* *_r_* = 646.65
Monoclinic, 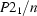

*a* = 12.7621 (14) Å
*b* = 9.2609 (11) Å
*c* = 21.683 (2) Å
β = 92.168 (2)°
*V* = 2560.9 (5) Å^3^
*Z* = 4
Mo *K*α radiation
μ = 1.79 mm^−1^
*T* = 298 K
0.48 × 0.42 × 0.21 mm

#### Data collection

Bruker SMART 1000 diffractometer
Absorption correction: multi-scan (*SADABS*; Sheldrick, 1996 ▶) *T*_min_ = 0.481, *T*_max_ = 0.706
12450 measured reflections
4504 independent reflections
2532 reflections with *I* > 2σ(*I*)
*R*_int_ = 0.064

#### Refinement

*R*[*F* ^2^ > 2σ(*F* ^2^)] = 0.046
*wR*(*F* ^2^) = 0.161
*S* = 1.00
4504 reflections
361 parameters
H-atom parameters constrained
Δρ_max_ = 0.48 e Å^−3^
Δρ_min_ = −0.42 e Å^−3^

### 

Data collection: *SMART* (Siemens, 1996 ▶); cell refinement: *SAINT* (Siemens, 1996 ▶); data reduction: *SAINT*; program(s) used to solve structure: *SHELXS97* (Sheldrick, 2008 ▶); program(s) used to refine structure: *SHELXL97* (Sheldrick, 2008 ▶); molecular graphics: *SHELXTL* (Sheldrick, 2008 ▶); software used to prepare material for publication: *SHELXTL*.

## Supplementary Material

Crystal structure: contains datablocks I, global. DOI: 10.1107/S1600536809040951/cv2616sup1.cif

Structure factors: contains datablocks I. DOI: 10.1107/S1600536809040951/cv2616Isup2.hkl

Additional supplementary materials:  crystallographic information; 3D view; checkCIF report

## Figures and Tables

**Table 1:** Hydrogen-bond geometry (Å, °)

*D*—H⋯*A*	*D*—H	H⋯*A*	*D*⋯*A*	*D*—H⋯*A*
C23—H23⋯N3^i^	0.93	2.53	3.451 (8)	173

Symmetry code: (i) 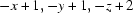.

**Fig. 1.**
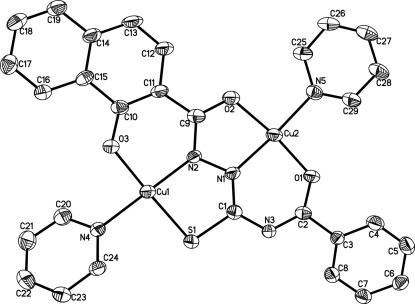
The molecular structure of the title compound, showing 30% probability displacement ellipsoids. H atoms have been omitted for clarity.

## supplementary crystallographic information

### Comment

Acylthiosemicarbazides possess strong coordination ability, and their complexes exhibit various biological activities (Wei *et al.*, 1995). Herewith we present the crystal structure of the title compound (I) - new dinuclear complex of naphthalenecarbothiosemicarbazide.

In (I) (Fig. 1), two Cu centers have different coordination environments - N_2_OS and N_2_O_2_, respectively, exhibiting distorted square-planar geometry each. The Cu1 atom is coordinated with one phenolate oxygen, one nitrogen atom of pyridine, one sulfur atom and one hydrazide nitrogen atom, forming five-membered and six-membered chelating rings. The Cu2 atom is coordinated with two carbonyl oxygen atoms, one nitrogen atom of pyridine and one hydrazide nitrogen atom, forming five-membered and six-membered chelating rings too. π–π interactions between the aromatic rings of neighbouring complexes [centroid-to centroid distance = 3.856 (5) Å] link two molecules into centrosymmetric dimers, which are further packed into stacks along *b* axis with relatively short Cu···Cu separation of 3.482 (1) Å. Weak intermolecular C—H···N hydrogen bonds (Table 1) help to consolidate the crystal packing.

### Experimental

1.406 g (10 mmol) of benzoyl chloride was added dropwise to a stirred solution of acetonitrile (50 ml) containing 0.981 g (10 mmol) of potassium thiocyanate. Stirring was continued for 1 h, and the solution was slowly warmed to ambient temperature. Then 1.68 g (10 mmol) of 1-hydroxy-2-naphthalenecarbohydrazide was added to the mixture, with stirring being continued for 5 h. After staying for overnight at refrigerator, the resulting yellow precipitate was filtered and rinsed with diethyl ether, then dried *in vacuo*, 87% yield. m.p. 225–227 C. The solution of CuNO_3_(0.04 g, 0.2 mmol) in pyridine(10 ml) was added to the mixture of *N*-benzoyl-1-hydroxy-2-naphthalenecarbothiosemicarbazide (0.073 g, 0.2 mmol)and sodium methylate(0.0324 g,0.6 mmol)in DMSO(10 ml). A green solution was obtained after refluxing for 3 h. After filtrated,dimethyl ether was slowly diffused into the filtrate, then crystals suitable for X-ray diffraction were obtained after two weeks (m.p. >400 K) (Chen *et al.* 2007) Elemental analysis calculated for Cu_2_C_29_H_21_N_5_O_3_S_1_: C, 53.64; H, 3.29; N, 9.43. Found (%): C, 53.86; H, 3.27; N, 10.83.

### Refinement

The C-bound H atoms were geometrically positioned (C—H = 0.93 Å), and were refined as riding, with *U*_iso_(H) = 1.2 *U*_eq_(C).

### Crystal data

**Table:**

[Cu_2_(C_19_H_11_N_3_O_3_S)(C_5_H_5_N)_2_]	*F*(000) = 1312
*M**_r_* = 646.65	*D*_x_ = 1.677 Mg m^−^^3^
Monoclinic, *P*2_1_/*n*	Mo *K*α radiation, λ = 0.71073 Å
*a* = 12.7621 (14) Å	Cell parameters from 2465 reflections
*b* = 9.2609 (11) Å	θ = 2.4–22.0°
*c* = 21.683 (2) Å	µ = 1.79 mm^−^^1^
β = 92.168 (2)°	*T* = 298 K
*V* = 2560.9 (5) Å^3^	Block, green
*Z* = 4	0.48 × 0.42 × 0.21 mm

### Data collection

**Table:**

Bruker SMART 1000 diffractometer	4504 independent reflections
Radiation source: fine-focus sealed tube	2532 reflections with *I* > 2σ(*I*)
graphite	*R*_int_ = 0.064
φ and ω scans	θ_max_ = 25.0°, θ_min_ = 1.8°
Absorption correction: multi-scan (*SADABS*; Sheldrick, 1996)	*h* = −15→15
*T*_min_ = 0.481, *T*_max_ = 0.706	*k* = −9→11
12450 measured reflections	*l* = −22→25

### Refinement

**Table:**

Refinement on *F*^2^	Primary atom site location: structure-invariant direct methods
Least-squares matrix: full	Secondary atom site location: difference Fourier map
*R*[*F*^2^ > 2σ(*F*^2^)] = 0.046	Hydrogen site location: inferred from neighbouring sites
*wR*(*F*^2^) = 0.161	H-atom parameters constrained
*S* = 1.00	*w* = 1/[σ^2^(*F*_o_^2^) + (0.0835*P*)^2^], where *P* = (*F*_o_^2^ + 2*F*_c_^2^)/3
4504 reflections	(Δ/σ)_max_ = 0.001
361 parameters	Δρ_max_ = 0.48 e Å^−^^3^
0 restraints	Δρ_min_ = −0.42 e Å^−^^3^

### Fractional atomic coordinates and isotropic or equivalent isotropic displacement parameters (Å^2^)

**Table:**

	*x*	*y*	*z*	*U*_iso_*/*U*_eq_	
Cu1	0.28704 (5)	0.20605 (8)	1.04261 (3)	0.0436 (3)	
Cu2	−0.01899 (6)	0.36338 (9)	0.94557 (3)	0.0496 (3)	
S1	0.33285 (12)	0.34418 (19)	0.96271 (7)	0.0508 (5)	
N1	0.1252 (4)	0.3405 (5)	0.9676 (2)	0.0431 (12)	
N2	0.1458 (4)	0.2438 (5)	1.0159 (2)	0.0416 (12)	
N3	0.1971 (4)	0.4823 (5)	0.8891 (2)	0.0418 (12)	
N4	0.4361 (4)	0.1750 (5)	1.0747 (2)	0.0432 (12)	
N5	−0.1717 (4)	0.3765 (6)	0.9268 (2)	0.0469 (13)	
O1	0.0142 (3)	0.4972 (5)	0.88313 (19)	0.0540 (12)	
O2	−0.0331 (3)	0.2309 (5)	1.0121 (2)	0.0585 (12)	
O3	0.2433 (3)	0.0804 (5)	1.10597 (19)	0.0526 (11)	
C1	0.2062 (5)	0.3912 (6)	0.9385 (3)	0.0415 (15)	
C2	0.1062 (5)	0.5270 (7)	0.8657 (3)	0.0446 (15)	
C3	0.1091 (5)	0.6286 (6)	0.8125 (3)	0.0407 (14)	
C4	0.0197 (5)	0.6999 (8)	0.7931 (3)	0.0595 (19)	
H4	−0.0429	0.6815	0.8122	0.071*	
C5	0.0213 (6)	0.7982 (8)	0.7456 (3)	0.067 (2)	
H5	−0.0396	0.8474	0.7337	0.080*	
C6	0.1120 (5)	0.8241 (7)	0.7158 (3)	0.0542 (18)	
H6	0.1135	0.8910	0.6839	0.065*	
C7	0.2003 (5)	0.7498 (8)	0.7339 (3)	0.0561 (18)	
H7	0.2620	0.7652	0.7134	0.067*	
C8	0.1998 (5)	0.6532 (7)	0.7815 (3)	0.0527 (17)	
H8	0.2609	0.6038	0.7931	0.063*	
C9	0.0565 (5)	0.1886 (7)	1.0356 (3)	0.0488 (16)	
C10	0.1489 (5)	0.0355 (7)	1.1163 (3)	0.0459 (16)	
C11	0.0576 (5)	0.0804 (7)	1.0840 (3)	0.0466 (16)	
C12	−0.0399 (5)	0.0174 (8)	1.0983 (3)	0.066 (2)	
H12	−0.0999	0.0467	1.0760	0.079*	
C13	−0.0496 (6)	−0.0831 (9)	1.1429 (3)	0.070 (2)	
H13	−0.1151	−0.1215	1.1507	0.084*	
C14	0.0390 (5)	−0.1290 (7)	1.1771 (3)	0.0552 (18)	
C15	0.1381 (5)	−0.0719 (7)	1.1649 (3)	0.0460 (16)	
C16	0.2265 (5)	−0.1219 (7)	1.1990 (3)	0.0511 (17)	
H16	0.2925	−0.0856	1.1908	0.061*	
C17	0.2165 (6)	−0.2236 (8)	1.2443 (3)	0.0607 (19)	
H17	0.2755	−0.2570	1.2665	0.073*	
C18	0.1178 (6)	−0.2766 (8)	1.2570 (3)	0.064 (2)	
H18	0.1110	−0.3431	1.2888	0.077*	
C19	0.0320 (6)	−0.2337 (8)	1.2244 (3)	0.0615 (19)	
H19	−0.0328	−0.2733	1.2329	0.074*	
C20	0.4648 (6)	0.0479 (8)	1.0975 (3)	0.0620 (19)	
H20	0.4152	−0.0257	1.0978	0.074*	
C21	0.5641 (6)	0.0195 (9)	1.1207 (4)	0.075 (2)	
H21	0.5816	−0.0723	1.1351	0.090*	
C22	0.6363 (6)	0.1260 (9)	1.1223 (3)	0.071 (2)	
H22	0.7040	0.1089	1.1380	0.085*	
C23	0.6084 (5)	0.2589 (8)	1.1005 (3)	0.0593 (19)	
H23	0.6562	0.3347	1.1019	0.071*	
C24	0.5086 (5)	0.2789 (7)	1.0765 (3)	0.0497 (16)	
H24	0.4905	0.3693	1.0606	0.060*	
C25	−0.2383 (5)	0.2922 (8)	0.9581 (3)	0.0553 (18)	
H25	−0.2114	0.2330	0.9896	0.066*	
C26	−0.3436 (5)	0.2916 (8)	0.9450 (3)	0.060 (2)	
H26	−0.3874	0.2313	0.9667	0.072*	
C27	−0.3842 (5)	0.3799 (9)	0.8998 (3)	0.063 (2)	
H27	−0.4559	0.3805	0.8904	0.075*	
C28	−0.3189 (5)	0.4669 (9)	0.8687 (3)	0.065 (2)	
H28	−0.3447	0.5290	0.8380	0.078*	
C29	−0.2128 (5)	0.4602 (8)	0.8839 (3)	0.0571 (18)	
H29	−0.1679	0.5190	0.8623	0.069*	

### Atomic displacement parameters (Å^2^)

**Table:**

	*U*^11^	*U*^22^	*U*^33^	*U*^12^	*U*^13^	*U*^23^
Cu1	0.0386 (4)	0.0450 (5)	0.0473 (5)	−0.0080 (4)	0.0043 (3)	0.0035 (4)
Cu2	0.0350 (4)	0.0602 (6)	0.0536 (5)	−0.0116 (4)	0.0012 (4)	0.0081 (4)
S1	0.0370 (9)	0.0626 (12)	0.0528 (10)	−0.0031 (8)	0.0045 (7)	0.0147 (8)
N1	0.038 (3)	0.044 (3)	0.047 (3)	−0.012 (2)	−0.001 (2)	0.008 (2)
N2	0.039 (3)	0.051 (3)	0.035 (3)	−0.010 (2)	0.000 (2)	0.011 (2)
N3	0.036 (3)	0.046 (3)	0.043 (3)	−0.005 (2)	0.000 (2)	0.009 (2)
N4	0.043 (3)	0.036 (3)	0.050 (3)	−0.003 (2)	0.002 (2)	0.005 (2)
N5	0.038 (3)	0.056 (4)	0.046 (3)	−0.006 (3)	−0.004 (3)	−0.005 (3)
O1	0.033 (2)	0.065 (3)	0.063 (3)	−0.012 (2)	−0.001 (2)	0.015 (2)
O2	0.042 (3)	0.075 (3)	0.059 (3)	−0.012 (2)	0.001 (2)	0.017 (2)
O3	0.038 (2)	0.061 (3)	0.059 (3)	−0.009 (2)	0.003 (2)	0.014 (2)
C1	0.043 (4)	0.038 (4)	0.043 (4)	−0.004 (3)	0.000 (3)	−0.005 (3)
C2	0.039 (4)	0.048 (4)	0.047 (4)	−0.009 (3)	0.001 (3)	−0.004 (3)
C3	0.038 (3)	0.042 (4)	0.041 (3)	−0.006 (3)	−0.005 (3)	0.004 (3)
C4	0.041 (4)	0.080 (5)	0.058 (4)	−0.003 (4)	0.006 (3)	0.011 (4)
C5	0.050 (4)	0.082 (6)	0.069 (5)	0.011 (4)	−0.003 (4)	0.019 (4)
C6	0.060 (5)	0.055 (5)	0.048 (4)	−0.010 (4)	−0.002 (3)	0.011 (3)
C7	0.044 (4)	0.068 (5)	0.056 (4)	−0.001 (4)	0.007 (3)	0.009 (4)
C8	0.046 (4)	0.059 (5)	0.053 (4)	0.001 (3)	−0.001 (3)	0.006 (3)
C9	0.042 (4)	0.060 (5)	0.045 (4)	−0.012 (3)	0.006 (3)	−0.003 (3)
C10	0.044 (4)	0.052 (4)	0.042 (4)	−0.013 (3)	0.003 (3)	−0.005 (3)
C11	0.044 (4)	0.053 (4)	0.043 (4)	−0.015 (3)	0.009 (3)	0.005 (3)
C12	0.044 (4)	0.087 (6)	0.067 (5)	−0.021 (4)	0.003 (3)	0.024 (4)
C13	0.052 (5)	0.091 (6)	0.069 (5)	−0.032 (4)	0.015 (4)	0.020 (4)
C14	0.054 (4)	0.053 (5)	0.059 (4)	−0.016 (4)	0.008 (4)	0.010 (3)
C15	0.060 (4)	0.042 (4)	0.037 (4)	−0.007 (3)	0.013 (3)	−0.003 (3)
C16	0.048 (4)	0.056 (5)	0.050 (4)	−0.005 (3)	0.008 (3)	0.005 (3)
C17	0.070 (5)	0.058 (5)	0.054 (4)	0.012 (4)	0.006 (4)	0.002 (4)
C18	0.079 (6)	0.057 (5)	0.057 (5)	0.000 (4)	0.026 (4)	0.012 (4)
C19	0.060 (5)	0.061 (5)	0.065 (5)	−0.016 (4)	0.011 (4)	0.008 (4)
C20	0.061 (5)	0.049 (5)	0.075 (5)	−0.008 (4)	−0.001 (4)	0.008 (4)
C21	0.067 (5)	0.050 (5)	0.107 (6)	0.010 (4)	−0.005 (5)	0.021 (4)
C22	0.047 (4)	0.079 (6)	0.087 (6)	0.007 (4)	−0.005 (4)	0.006 (5)
C23	0.048 (4)	0.061 (5)	0.068 (5)	−0.016 (4)	−0.005 (4)	0.006 (4)
C24	0.045 (4)	0.046 (4)	0.059 (4)	−0.003 (3)	0.011 (3)	0.011 (3)
C25	0.045 (4)	0.072 (5)	0.050 (4)	−0.013 (4)	0.011 (3)	−0.003 (4)
C26	0.048 (4)	0.081 (6)	0.053 (4)	−0.022 (4)	0.016 (3)	−0.011 (4)
C27	0.039 (4)	0.088 (6)	0.062 (5)	−0.014 (4)	0.006 (4)	−0.022 (4)
C28	0.036 (4)	0.093 (6)	0.067 (5)	−0.010 (4)	−0.005 (4)	−0.009 (4)
C29	0.039 (4)	0.073 (5)	0.060 (4)	−0.010 (4)	0.005 (3)	−0.002 (4)

### Geometric parameters (Å, °)

**Table:**

Cu1—O3	1.900 (4)	C10—C15	1.460 (8)
Cu1—N2	1.905 (5)	C11—C12	1.420 (8)
Cu1—N4	2.022 (5)	C12—C13	1.350 (9)
Cu1—S1	2.2487 (17)	C12—H12	0.9300
Cu2—O1	1.895 (4)	C13—C14	1.395 (9)
Cu2—N1	1.896 (5)	C13—H13	0.9300
Cu2—O2	1.908 (4)	C14—C15	1.405 (8)
Cu2—N5	1.980 (5)	C14—C19	1.415 (9)
S1—C1	1.736 (6)	C15—C16	1.404 (8)
N1—C1	1.317 (7)	C16—C17	1.370 (9)
N1—N2	1.395 (6)	C16—H16	0.9300
N2—C9	1.333 (7)	C17—C18	1.390 (9)
N3—C2	1.314 (7)	C17—H17	0.9300
N3—C1	1.366 (7)	C18—C19	1.341 (9)
N4—C20	1.322 (8)	C18—H18	0.9300
N4—C24	1.335 (7)	C19—H19	0.9300
N5—C29	1.305 (8)	C20—C21	1.372 (9)
N5—C25	1.355 (8)	C20—H20	0.9300
O1—C2	1.278 (7)	C21—C22	1.349 (10)
O2—C9	1.295 (7)	C21—H21	0.9300
O3—C10	1.302 (7)	C22—C23	1.361 (10)
C2—C3	1.490 (8)	C22—H22	0.9300
C3—C4	1.372 (8)	C23—C24	1.371 (8)
C3—C8	1.379 (8)	C23—H23	0.9300
C4—C5	1.375 (9)	C24—H24	0.9300
C4—H4	0.9300	C25—C26	1.363 (9)
C5—C6	1.368 (9)	C25—H25	0.9300
C5—H5	0.9300	C26—C27	1.364 (10)
C6—C7	1.365 (8)	C26—H26	0.9300
C6—H6	0.9300	C27—C28	1.356 (9)
C7—C8	1.367 (9)	C27—H27	0.9300
C7—H7	0.9300	C28—C29	1.384 (8)
C8—H8	0.9300	C28—H28	0.9300
C9—C11	1.451 (8)	C29—H29	0.9300
C10—C11	1.400 (8)		
			
O3—Cu1—N2	91.84 (18)	C11—C10—C15	117.6 (5)
O3—Cu1—N4	87.81 (18)	C10—C11—C12	119.3 (6)
N2—Cu1—N4	176.7 (2)	C10—C11—C9	123.5 (5)
O3—Cu1—S1	175.88 (14)	C12—C11—C9	117.2 (6)
N2—Cu1—S1	86.09 (14)	C13—C12—C11	122.9 (6)
N4—Cu1—S1	94.46 (14)	C13—C12—H12	118.5
O1—Cu2—N1	90.70 (19)	C11—C12—H12	118.5
O1—Cu2—O2	172.29 (18)	C12—C13—C14	119.8 (6)
N1—Cu2—O2	81.73 (19)	C12—C13—H13	120.1
O1—Cu2—N5	93.3 (2)	C14—C13—H13	120.1
N1—Cu2—N5	176.0 (2)	C13—C14—C15	120.1 (6)
O2—Cu2—N5	94.3 (2)	C13—C14—C19	121.5 (6)
C1—S1—Cu1	96.3 (2)	C15—C14—C19	118.4 (6)
C1—N1—N2	117.4 (5)	C16—C15—C14	119.2 (6)
C1—N1—Cu2	127.7 (4)	C16—C15—C10	120.6 (6)
N2—N1—Cu2	114.4 (3)	C14—C15—C10	120.2 (6)
C9—N2—N1	110.4 (5)	C17—C16—C15	120.6 (6)
C9—N2—Cu1	129.9 (4)	C17—C16—H16	119.7
N1—N2—Cu1	119.7 (3)	C15—C16—H16	119.7
C2—N3—C1	123.0 (5)	C16—C17—C18	119.6 (7)
C20—N4—C24	116.7 (6)	C16—C17—H17	120.2
C20—N4—Cu1	119.9 (4)	C18—C17—H17	120.2
C24—N4—Cu1	123.3 (4)	C19—C18—C17	121.2 (7)
C29—N5—C25	117.2 (6)	C19—C18—H18	119.4
C29—N5—Cu2	123.2 (4)	C17—C18—H18	119.4
C25—N5—Cu2	119.5 (5)	C18—C19—C14	120.9 (7)
C2—O1—Cu2	125.8 (4)	C18—C19—H19	119.6
C9—O2—Cu2	112.6 (4)	C14—C19—H19	119.6
C10—O3—Cu1	128.3 (4)	N4—C20—C21	123.1 (7)
N1—C1—N3	123.4 (5)	N4—C20—H20	118.4
N1—C1—S1	120.3 (5)	C21—C20—H20	118.4
N3—C1—S1	116.3 (4)	C22—C21—C20	119.4 (7)
O1—C2—N3	128.8 (6)	C22—C21—H21	120.3
O1—C2—C3	114.5 (5)	C20—C21—H21	120.3
N3—C2—C3	116.7 (5)	C21—C22—C23	118.8 (7)
C4—C3—C8	118.3 (6)	C21—C22—H22	120.6
C4—C3—C2	119.8 (6)	C23—C22—H22	120.6
C8—C3—C2	121.8 (6)	C22—C23—C24	118.8 (7)
C3—C4—C5	120.9 (6)	C22—C23—H23	120.6
C3—C4—H4	119.6	C24—C23—H23	120.6
C5—C4—H4	119.6	N4—C24—C23	123.1 (6)
C6—C5—C4	120.5 (7)	N4—C24—H24	118.4
C6—C5—H5	119.8	C23—C24—H24	118.4
C4—C5—H5	119.8	N5—C25—C26	122.0 (7)
C7—C6—C5	118.7 (6)	N5—C25—H25	119.0
C7—C6—H6	120.7	C26—C25—H25	119.0
C5—C6—H6	120.7	C25—C26—C27	119.5 (7)
C6—C7—C8	121.4 (6)	C25—C26—H26	120.2
C6—C7—H7	119.3	C27—C26—H26	120.2
C8—C7—H7	119.3	C28—C27—C26	119.3 (7)
C7—C8—C3	120.3 (6)	C28—C27—H27	120.3
C7—C8—H8	119.9	C26—C27—H27	120.3
C3—C8—H8	119.9	C27—C28—C29	117.9 (7)
O2—C9—N2	120.7 (6)	C27—C28—H28	121.0
O2—C9—C11	118.5 (5)	C29—C28—H28	121.0
N2—C9—C11	120.8 (6)	N5—C29—C28	124.0 (7)
O3—C10—C11	125.2 (6)	N5—C29—H29	118.0
O3—C10—C15	117.1 (6)	C28—C29—H29	118.0
			
O3—Cu1—S1—C1	63.1 (19)	C3—C4—C5—C6	1.7 (11)
N2—Cu1—S1—C1	3.2 (2)	C4—C5—C6—C7	0.4 (11)
N4—Cu1—S1—C1	−173.5 (2)	C5—C6—C7—C8	−1.3 (11)
O1—Cu2—N1—C1	−8.4 (5)	C6—C7—C8—C3	0.0 (11)
O2—Cu2—N1—C1	173.0 (6)	C4—C3—C8—C7	2.0 (10)
N5—Cu2—N1—C1	167 (3)	C2—C3—C8—C7	−178.4 (6)
O1—Cu2—N1—N2	−179.8 (4)	Cu2—O2—C9—N2	4.1 (8)
O2—Cu2—N1—N2	1.6 (4)	Cu2—O2—C9—C11	−176.1 (5)
N5—Cu2—N1—N2	−5(3)	N1—N2—C9—O2	−2.8 (8)
C1—N1—N2—C9	−172.4 (5)	Cu1—N2—C9—O2	178.5 (4)
Cu2—N1—N2—C9	0.0 (6)	N1—N2—C9—C11	177.5 (5)
C1—N1—N2—Cu1	6.5 (7)	Cu1—N2—C9—C11	−1.2 (9)
Cu2—N1—N2—Cu1	178.9 (2)	Cu1—O3—C10—C11	−4.5 (9)
O3—Cu1—N2—C9	−3.5 (6)	Cu1—O3—C10—C15	174.4 (4)
N4—Cu1—N2—C9	−87 (3)	O3—C10—C11—C12	177.1 (6)
S1—Cu1—N2—C9	172.9 (6)	C15—C10—C11—C12	−1.9 (9)
O3—Cu1—N2—N1	177.9 (4)	O3—C10—C11—C9	−2.4 (11)
N4—Cu1—N2—N1	94 (3)	C15—C10—C11—C9	178.6 (6)
S1—Cu1—N2—N1	−5.7 (4)	O2—C9—C11—C10	−174.5 (6)
O3—Cu1—N4—C20	34.8 (5)	N2—C9—C11—C10	5.3 (10)
N2—Cu1—N4—C20	119 (3)	O2—C9—C11—C12	6.0 (9)
S1—Cu1—N4—C20	−141.7 (5)	N2—C9—C11—C12	−174.2 (6)
O3—Cu1—N4—C24	−142.7 (5)	C10—C11—C12—C13	1.3 (12)
N2—Cu1—N4—C24	−59 (3)	C9—C11—C12—C13	−179.2 (7)
S1—Cu1—N4—C24	40.7 (5)	C11—C12—C13—C14	0.0 (12)
O1—Cu2—N5—C29	−0.9 (5)	C12—C13—C14—C15	−0.6 (12)
N1—Cu2—N5—C29	−176 (3)	C12—C13—C14—C19	179.8 (7)
O2—Cu2—N5—C29	177.8 (5)	C13—C14—C15—C16	−178.6 (7)
O1—Cu2—N5—C25	177.0 (5)	C19—C14—C15—C16	1.0 (10)
N1—Cu2—N5—C25	2(3)	C13—C14—C15—C10	−0.1 (10)
O2—Cu2—N5—C25	−4.3 (5)	C19—C14—C15—C10	179.6 (6)
N1—Cu2—O1—C2	7.9 (5)	O3—C10—C15—C16	0.9 (9)
O2—Cu2—O1—C2	18.4 (18)	C11—C10—C15—C16	179.9 (6)
N5—Cu2—O1—C2	−171.8 (5)	O3—C10—C15—C14	−177.7 (6)
O1—Cu2—O2—C9	−13.7 (18)	C11—C10—C15—C14	1.3 (9)
N1—Cu2—O2—C9	−3.0 (4)	C14—C15—C16—C17	−0.9 (10)
N5—Cu2—O2—C9	176.6 (4)	C10—C15—C16—C17	−179.5 (6)
N2—Cu1—O3—C10	6.4 (5)	C15—C16—C17—C18	−0.7 (10)
N4—Cu1—O3—C10	−176.9 (5)	C16—C17—C18—C19	2.3 (11)
S1—Cu1—O3—C10	−53 (2)	C17—C18—C19—C14	−2.2 (11)
N2—N1—C1—N3	177.5 (5)	C13—C14—C19—C18	−179.8 (7)
Cu2—N1—C1—N3	6.3 (9)	C15—C14—C19—C18	0.5 (11)
N2—N1—C1—S1	−3.0 (8)	C24—N4—C20—C21	−1.6 (10)
Cu2—N1—C1—S1	−174.2 (3)	Cu1—N4—C20—C21	−179.3 (6)
C2—N3—C1—N1	−0.4 (9)	N4—C20—C21—C22	1.9 (12)
C2—N3—C1—S1	180.0 (5)	C20—C21—C22—C23	−0.3 (12)
Cu1—S1—C1—N1	−1.1 (5)	C21—C22—C23—C24	−1.4 (11)
Cu1—S1—C1—N3	178.5 (4)	C20—N4—C24—C23	−0.2 (9)
Cu2—O1—C2—N3	−5.5 (10)	Cu1—N4—C24—C23	177.4 (5)
Cu2—O1—C2—C3	175.8 (4)	C22—C23—C24—N4	1.7 (10)
C1—N3—C2—O1	0.1 (10)	C29—N5—C25—C26	1.4 (10)
C1—N3—C2—C3	178.8 (5)	Cu2—N5—C25—C26	−176.7 (5)
O1—C2—C3—C4	10.6 (9)	N5—C25—C26—C27	−1.2 (11)
N3—C2—C3—C4	−168.2 (6)	C25—C26—C27—C28	0.0 (11)
O1—C2—C3—C8	−168.9 (6)	C26—C27—C28—C29	0.9 (11)
N3—C2—C3—C8	12.2 (9)	C25—N5—C29—C28	−0.4 (10)
C8—C3—C4—C5	−2.9 (10)	Cu2—N5—C29—C28	177.6 (5)
C2—C3—C4—C5	177.5 (6)	C27—C28—C29—N5	−0.7 (11)

### Hydrogen-bond geometry (Å, °)

**Table:**

*D*—H···*A*	*D*—H	H···*A*	*D*···*A*	*D*—H···*A*
C23—H23···N3^i^	0.93	2.53	3.451 (8)	173

Symmetry codes: (i) −*x*+1, −*y*+1, −*z*+2.
